# Specific interpretation biases as a function of social anxiety and callous-unemotional traits in a community and a clinical adolescent sample

**DOI:** 10.1186/s13034-023-00585-z

**Published:** 2023-03-31

**Authors:** Anna L. Dapprich, Eni S. Becker, Laura M. Derks, Tanja Legenbauer, Wolf-Gero Lange

**Affiliations:** 1 Behavioural Science Institute, Radboud University, 500 HB Nijmegen, The Netherlands; 2 LWL-University Hospital Hamm for Child and Adolescent Psychiatry, Psychotherapy and Psychosomatics, Ruhr-University Bochum, Bochum, Germany

**Keywords:** Internalizing, Externalizing, Anxiety, Aggression, Cognitive biases, Social information processing, Psychopathology

## Abstract

**Background:**

Threatening and hostile interpretation biases are seen as causal and maintaining mechanisms of childhood anxiety and aggression, respectively. However, it is unclear whether these interpretation biases are specific to distinct problems or whether they are general psychopathological phenomena. The specificity versus pervasiveness of interpretation biases could also differ depending on mental health status. Therefore, in the current study, we investigated whether social anxiety and callous-unemotional (CU) traits were uniquely related to threatening and hostile interpretation biases, respectively, in both a community and a clinical sample of adolescents.

**Methods:**

A total of 161 adolescents between 10 to 15 years of age participated. The community sample consisted of 88 participants and the clinical sample consisted of 73 inpatients with a variety of psychological disorders. Social anxiety and CU-traits were assessed with self-report questionnaires. The Ambiguous Social Scenario Task was used to measure both threatening and hostile interpretations in response to written vignettes.

**Results:**

Results showed that social anxiety was uniquely related to more threatening interpretations, while CU-traits were uniquely related to more hostile interpretations. These relationships were replicated for the community sample. For the clinical sample, only the link between social anxiety and threatening interpretations was significant. Explorative analyses showed that adolescents with externalizing disorders scored higher on hostile interpretations than adolescents with internalizing disorders.

**Conclusions:**

Overall, these results support the content-specificity of threatening interpretation biases in social anxiety and of hostile interpretation biases in CU-traits. Better understanding the roles of interpretation biases in different psychopathologies might open avenues for tailored prevention and intervention paradigms.

Many theories stress the role of interpretation biases, i.e. systematic distortions in the interpretation of environmental cues, in problematic behavior and psychopathology throughout childhood and adolescence [1–3]. Negative, threatening interpretations have mostly been linked to internalizing problems, such as depression and anxiety (for review, see [4–6]), whereas hostile interpretations have mostly been linked to externalizing problems, such as aggression and conduct problems (for review, see [7–9]). However, some studies found that children with elevated anxiety levels also show hostile interpretations [10, 11], and that aggressive children also show threatening interpretations [12]. These seemingly contrasting findings raise the question of whether interpretation biases are specific to particular problems or disorders (i.e. *content-specificity hypothesis*; [13, 14]), or whether they are general and a commonality between anxiety and aggression [15–17]. The specificity versus generalizability of interpretation biases could also depend on an individual’s mental health status and the presence of psychopathology [5]. To better understand the occurrences of interpretation biases and their role in the development of psychopathology, it is necessary to investigate their link to both internalizing and externalizing problems in samples with varying levels of psychopathology. Therefore, the present study investigates whether threatening and hostile interpretation biases of identical social situations are specific to self-reported social anxiety and callous-unemotional (CU) traits in a clinical and a community sample of children.

Social anxiety and CU-traits are relevant concepts in internalizing and externalizing problems, respectively, and they are common in community samples [18, 19]. Social anxiety is characterized by high fear in and the avoidance of social situations in which devaluation by others is possible [20]. CU-traits are characterized by the lack of guilt and remorse, flat affect and disinterest in important activities such as school and social relationships [21, 22]. Increased levels of social anxiety and CU-traits are seen as normative during adolescence [23–25]. However, social anxiety disorder typically develops during adolescence [26], which increases the risk on comorbid depression, other anxiety disorders and substance abuse [20]. Furthermore, CU-traits in adolescence are related to severe, persistent aggressive behavior, as well as a higher risk on the development of antisocial personality disorder and psychopathy later in life [27]. Causal and maintaining mechanisms of social anxiety and aggressive behavior are threatening [3, 28, 29] and hostile interpretation biases [1, 7, 8, 30, 31], respectively. Yet, there are some open questions regarding the link between interpretation biases and both social anxiety and CU-traits in adolescence.

The content-specificity of threatening interpretation bias has generally been supported for social anxiety (for reviews, see [4, 5]). For instance, while children with higher levels of social anxiety interpret social scenarios as the most threatening, children with higher levels of separation anxiety interpret separation scenarios as the most threatening ([32, 33]; for contrasting findings, see [34]). Furthermore, in both clinical and community samples the strength of threatening interpretation bias has been found to increase as the severity of social anxiety increases [33, 35–39]. However, only a few studies compared clinical to subclinical or non-clinical samples (for research that did so, see 33, 39). As a consequence, a meta-analysis concluded that the content-specificity of interpretation biases in childhood anxiety could be supported for different samples separately, but not across different samples [5]. To further support the content-specificity of interpretation biases, not only different samples but also different interpretations should be compared.

Research on interpretation biases in CU-traits examined different interpretation biases but the findings are inconsistent. Hostile interpretation bias has been found to be higher [40] or lower [41] as a function of CU-traits in community samples. Other studies did not find any link between CU-traits and hostile interpretation bias, neither in community nor in clinical samples [42, 43]. Threatening interpretation bias has been found to be increased in delinquent adolescents with higher levels of CU-traits [44]. Given the high comorbidity of internalizing and externalizing problems [45], controlling for both sort of problems might help to disentangle the relationships between CU-traits and interpretation biases.

The assessment of threatening and hostile interpretation biases together has shown support for content-specific interpretation biases in relation to social anxiety and CU-traits, respectively. The Ambiguous Social Scenario Task (ASST) is a newly developed task that measures both threatening and hostile interpretations in response to the same situations [46]. In a clinical sample of adolescent inpatients, self-reported social anxiety was uniquely related to threatening interpretations, and CU-traits were uniquely related to hostile interpretations [47]. By investigating whether these relationships express themselves similarly in different samples, the role of interpretation biases in different problems might be better understood.

The goal of the current study was to examine whether threatening and hostile interpretation biases are specific to social anxiety and CU-traits, respectively, in both a clinical and a community sample of adolescents. In line with the content-specificity of interpretation biases, we expected that higher levels of social anxiety were related to more threatening interpretations (e.g. [33]), and that higher levels of CU-traits were related to more hostile interpretations [47]. General, pervasive interpretation biases would be supported when social anxiety would be related to more hostile interpretations and when CU-traits were related to more threatening interpretations. We expected that the clinical sample, compared to the community sample, would score higher on both interpretation biases (as has already been shown for threatening interpretations; [33]), but we did not expect that the link between social anxiety, CU-traits and interpretation biases would differ between samples.

## Method

### Participants

For the current study, an existing clinical sample (fully described in 47) was matched to a newly recruited community sample. The existing clinical sample consisted of 401 participants (253 girls) between 10 and 20 years of age (M = 14.66, SD = 1.94). The three most common primary diagnoses based on the 10^th^ version of the International Classification of Diseases (ICD-10) were major depressive disorder (F32.-; n = 33; 45%), mixed disorder of conduct and emotion (F92.-; n = 25; 34%), and other emotional disorders (F93.-; n = 5; 9%). The newly recruited community sample consisted of 116 children and adolescents (76 girls) between 7 and 15 years of age (M = 11.09, SD = 1.93).

We randomly matched the number of boys and girls per age from the clinical sample with the number of boys and girls per age of the community sample by using the function *sample* of the R *base* package [48]. Since number of boys and girls was not always equally represented, the size and gender distribution differed for the two newly created samples. That is, the clinical sample consisted of 73 participants (44 girls) and the community sample consisted of 88 participants (58 girls). Both samples were between 10 and 15 years of age with a mean age of 12 years. The sample characteristics are shown in Table 1.

**Table 1:** Descriptive statistics of all variables per sample

	Community sample (N = 88, 58 girls)		Clinical sample (N = 73, 44 girls)		
	M (SD)	range	M (SD)	range	*p*
Age	11.9 (1.3)	10–15	12.3 (1.2)	10–15	n.s
Social anxiety	6.6 (3.6)	0–16	9.4 (5.6)	0–18	< .001
CU-traits	28.8 (8.6)	14–50	27.1 (8.9)	11–51	n.s
Threatening interpretations	38.2 (19.1)	2–87	51.1 (21.5)	0–98	< .001
Hostile interpretations	32.4 (16.0)	0–76	35.4 (18.4)	0–76	n.s
Neutral interpretations	46.4 (17.2)	5–92	39.7 (17.1)	5–82	< .05

*CU* callous-unemotional

### Measurements

#### Ambiguous Social Scenario Task (ASST—youth version; [46])

The ASST—youth version assesses hostile, threatening and neutral interpretations of ambiguous social situations. Participants have to indicate for 10 social scenarios how likely a threatening, a hostile and a neutral interpretation would come to their minds. An example situation is ‘You asked a question in a WhatsApp group. After a while you see that everyone read your message but no one responded.' The three interpretations given read 1) ‘Probably they find my question stupid and annoying.’ (threatening), 2) ‘People are lazy. I won’t answer their questions anymore, either.’ (hostile) and 3) ‘Probably everyone is busy and doesn’t have time to answer.’ (neutral). Answers are given for each interpretation on a visual analogue scale ranging from 0% (‘very unlikely’) to 100% (‘very likely’). For the analyses, mean scores for each interpretation category are used. The current internal consistencies were good for threatening interpretations (clinical sample: *α* = 0.83, community sample: *α* = 0.80), and acceptable for both hostile (clinical sample: *α* = 0.75, community sample: *α* = 0.71) and neutral interpretations (clinical sample: *α* = 0.73, community sample: *α* = 0.74).

#### Spence children’s anxiety scale (SCAS-D; [49])

The SCAS-D measures self-reported anxiety using six subscales that assess symptoms of social anxiety, panic disorder, agoraphobia, generalized anxiety disorder, obsessive–compulsive disorder, separation anxiety disorder, and specific phobia. Participants answer 38 items, such as ‘I feel afraid that I will make a fool of myself in front of people.‘ (social anxiety subscale), on a 4-point Likert scale ranging from 0 (‘never’) to 3 (‘always’). The social anxiety subscale consists of 6 items. For girls and boys, scores higher than 9 and 7, respectively, are considered as clinically-relevant (based on the cutoff scores published online; https://www.scaswebsite.com). The social anxiety subscale had an excellent internal consistency in the current study (clinical sample: *α* = 0.88, community sample: *α* = 0.77).

#### Inventory of callous-unemotional traits (ICU; [50])

The ICU assesses callous-unemotional (CU) traits in terms of three subscales, namely callousness (e.g., ‘I do not care who I hurt to get what I want’), uncaring (e.g., ‘I care about how well I do at school’, reversed score) and unemotional (e.g., ‘I do not show my emotions to others’). Participants rate 24 items on a 4-point Likert scale ranging from 0 (‘not at all true’) to 3 (‘definitely true’). Scores higher than 30 and 35 indicate at-risk for girls and boys, respectively (based on the cutoff scores published online; https://faculty.lsu.edu/pfricklab/icu.php). In the current study, the total ICU score had an acceptable internal consistency in the clinical sample (*α* = 0.74) and a slightly lower internal consistency in the community sample (*α* = 0.67). The internal consistencies of both the callousness and the uncaring subscales were in a similar range (clinical sample: *α* = 0.67 and 72, community sample: *α* = 0.57 and 60). However, the internal consistency of the unemotional subscale was low (clinical sample: *α* = 0.25, community sample: *α* = 0.27).

### Procedure

The community sample was recruited in German elementary and high schools. The schools distributed information about the study among parents. When parents indicated their interest in letting their children participate, they received a digital information letter and were asked for digital consent. After agreeing to their child’s participation, a link to the questionnaires was sent. Information letter, informed consent and questionnaires were provided by using the Qualtrics online platform [51]. It was stressed that children should fill in the questionnaires on their own by using a computer and by sitting in a quiet environment. Filling in the questionnaires took about 20 min. Participation was rewarded with a 5€ voucher. The ethical committee of the Faculty of Social Sciences had no formal objection to the study (ECSW-2020-154).

Data from the clinical sample were gathered as part of the diagnostic routine of an inpatient clinic within a predefined period of 6 months (for details see [47]). Informed consent was obtained from the patients’ legal guardians. The adolescents were verbally informed about the study and were given the possibility to refrain from participation. The whole diagnostic routine took approximately 1 h. The clinical sample did not receive a compensation for their participation. The local medical-ethical committee approved the use of the data (No.: 4359–12).

### Statistical approach

The main research questions, whether threatening and hostile interpretation biases are content-specific to social anxiety and CU-traits, respectively, and whether these links differ between a clinical and a community sample were examined by using multivariate multiple regression followed-up by univariate multiple regression. The relationships between all variables of interest were furthermore investigated by performing Pearson’s correlations and t-tests.

The freely available software R (version 4.0.3; [48]) and RStudio (RStudio 2022.02.3; [52]) were used for data preparation and analyses. The function *lm* of the *stats* package (version 4.0.3; [48]) was used to conduct both multivariate and univariate multiple regression. For multivariate multiple regression, both threatening and hostile interpretations were entered as outcome. Social anxiety, CU-traits, sample, as well as the two-way interactions between social anxiety and sample and between CU-traits and sample were entered as predictors. Univariate regression analyses were used to further interpret the results with only one interpretation bias at a time as outcome. Continuous predictors were standardized to align their scales. The correlations between all measurement were computed per sample by using the function *corr.test* of the package *psych* (version 2.0.12; [53]). Holm adjustment was used to control for multiple testing. The differences between the samples were further examined by performing t-tests using the function *t.test* of the *stats* package (version 4.0.3; [48]).

### Transparency statement

The current study has been pre-registered on osf.io (https://osf.io/jermg). Different to what was pre-registered, we used multivariate regression instead of the pre-registered Structural Equation Model (SEM). For SEM, a minimum of 300 participants is generally recommended [54]. Unfortunately, we did not achieve this sample size since the corona pandemic hindered our data collection resulting in time constraints. With a final sample size of 161 participants, five predictors and *p* = 0.05, we had an excellent power (1 − *ß* = 0.98) to detect effects of medium size (*f*^*2*^ = 0.15) with regression analyses. Therefore, we decided to analyze our principal research question with multivariate regression.

## Results

### The role of social anxiety, CU-traits and sample on both interpretation biases

Multivariate multiple regression analysis indicated significant main effects for both CU-traits, *V* = 0.08, *F*(2, 150) = 6.03, *p* = 0.003, and social anxiety, *V* = 0.15, *F*(2, 150) = 13.37, *p* < 0.001, on threatening and hostile interpretations together. The effect of sample was not significant. This indicates that both CU-traits and social anxiety play a role on interpretation biases. Next, we inspected the results of the univariate regression analyses for hostile and threatening interpretations separately.

#### The role of social anxiety, CU-traits and sample on threatening interpretations

The univariate multiple regression for threatening interpretations was significant *F*(5, 151) = 21.33, *p* < 0.001 with the predictors explaining 39.46% of the variance, 95% bootstrapped CI [30.60, 51.63]. A significant main effect of sample was found (*β* = 0.15, *p* = 0.02). A follow-up t-test showed that the clinical sample scored higher on both social anxiety, *t*(159) = -3.92, *p* < 0.001, and threatening interpretations than the community sample, *t*(159) = -4.04, *p* < 0.001 (see Table 1). Furthermore, a significant main effect for social anxiety on threatening interpretations was found (*β* = 0.60, *p* < 0.001). Pearson’s correlations indicated that higher levels of social anxiety were related to more threatening interpretations in both the clinical (*r* = 0.64) and the community sample (*r* = 0.44, both *p*’s < 0.001; see Table 2).

**Table 2:** Correlations between all measurements per sample

	Community sample				Clinical sample			
	Social anxiety	CU-traits	Threatening interpretations	Hostile interpretations	Social anxiety	CU-traits	Threatening interpretations	Hostile interpretations
CU-traits	− .13				− .01			
Threatening interpretations	.44***	.11			.66***	− .02		
Hostile interpretations	.11	.37**	.51***		− .19	.22	.05	
Neutral interpretations	− .19	.04	− .06	.09	− .34*	− .08	− .16	.35*

Holm adjustment was used to control for multiple testing

*CU* callous-unemotional

***p < .001; **p < .01; *p < .05

#### The role of social anxiety, CU-traits and sample on hostile interpretations

**Fig. 1:**
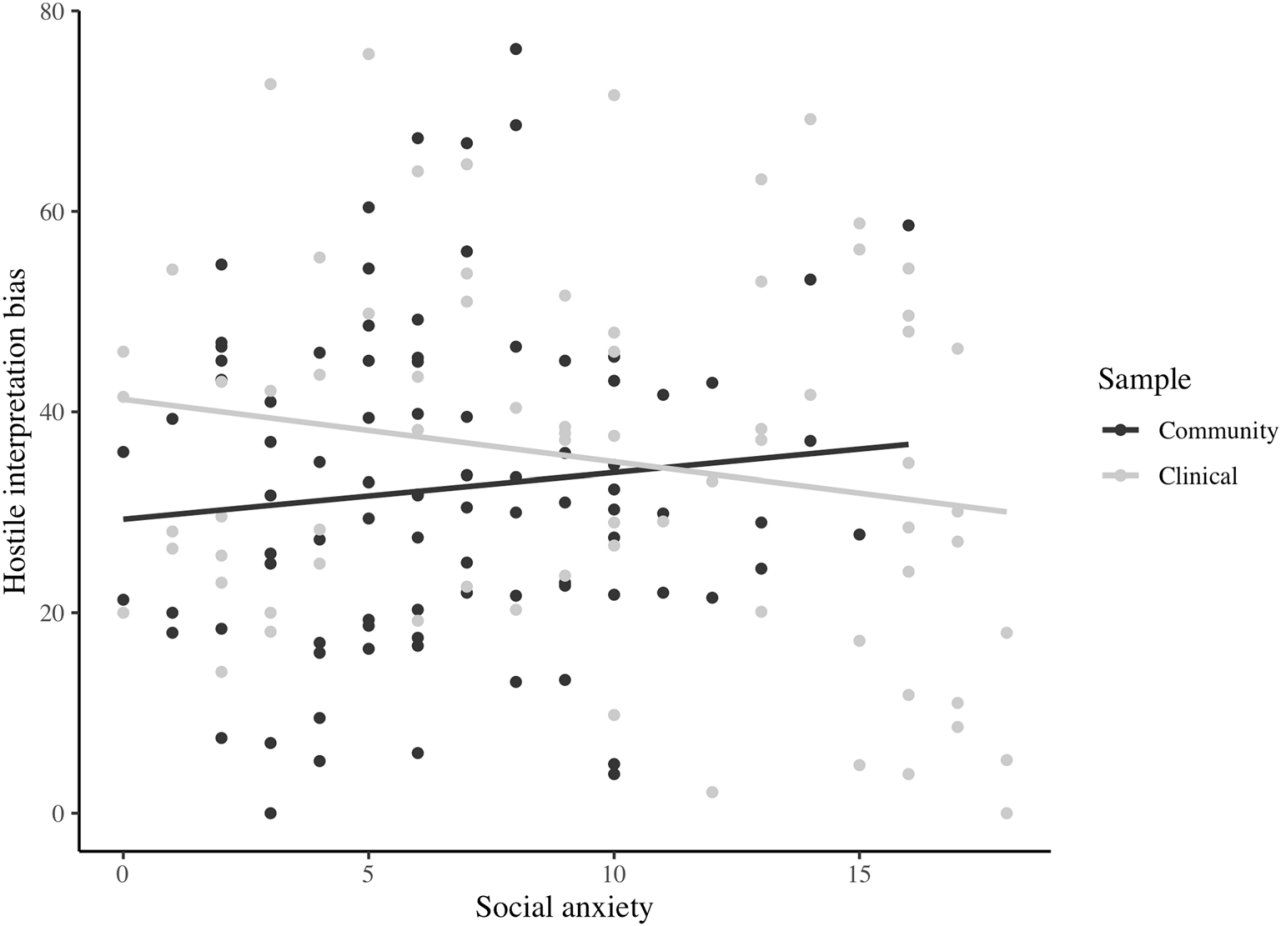
Significant interaction effect between social anxiety and sample on hostile interpretation bias

The univariate model for hostile interpretations was significant *F*(5, 151) = 4.42, *p* < 0.001 with the predictors explaining 9.87% of the variance, 95% bootstrapped CI [5.22, 20.42]. A significant interaction effect for social anxiety and sample on hostile interpretations was found (*β* = − 0.31, *p* = 0.03). Higher levels of social anxiety were related to more hostile interpretations in the community sample, but they were related to less hostile interpretations in the clinical sample (see Fig. 1). The correlations between social anxiety and hostile interpretations for each sample separately were not significant, though (see Table 2).

Furthermore, the results showed a significant main effect for CU-traits on hostile interpretations (*β* = 0.36, *p* < 0.001). For consistency, we also conducted t-tests for sample differences and Pearson’s correlation for each sample separately. Neither CU-traits nor hostile interpretations differed between the samples (both *p*’s > 0.05, see Table 1). Only for the community sample, a significant positive correlation between CU-traits and hostile interpretations was found (*r* = 0.37, *p* < 0.01). In the clinical sample, the relationship was positive but not significant (*r* = 0.22, *p* = 0.46; see Table 2).

### Exploring internalizing and externalizing diagnoses to differentiate interpretation biases

Given the unexpected finding that hostile interpretations were not related to CU-traits in the clinical sample, we explored whether grouping participants into externalizing and internalizing disorders would relate to hostile and threatening interpretation biases, respectively. The externalizing group consisted of 33 patients (10 girls). Most of them had a subtype of conduct disorders as main diagnosis (F90.1, F91.2, F92.0 and F92.8); Other diagnoses were addiction and attachment disorders (F12.2, F94.1 and F94.2). The internalizing group consisted of 39 patients (33 girls) including the main diagnoses depressive disorder (F32.,-), somatization disorder (F45.0) and other childhood emotional disorder (F93.8). One patient with the diagnosis atypical anorexia nervosa (F50.1) was not classified as internalizing or externalizing.

A 2 (externalizing disorders, internalizing disorders) group × 3 (hostile, threatening, neutral) interpretation repeated measures ANOVA on strength of interpretation yielded a significant main effect of interpretation, *F*(1.82, 127.7) = 13.2, *p* < 0.001, as well as a significant interaction effect for group and content of interpretation, *F*(1.82, 127.7) = 12.9, *p* < 0.001, on strength of interpretation bias. Follow-up t-tests for the interaction effect showed that threatening interpretations were higher in the internalizing group *t*(70) = − 3.23, *p* < 0.01, whereas hostile interpretations were higher in the externalizing group, *t*(70) = 2.15, *p* < 0.05. Furthermore, social anxiety was higher in the internalizing group, *t*(70) = − 5.29, p < 0.001, while CU-traits did not differ per group (*p* > 0.05).

### Exploring the relationships between interpretation biases

Pearson’s correlations indicated a significant positive correlation between threatening interpretations and hostile interpretations for the community sample (*r* = 0.51, *p* < 0.001). This correlation was not significant for the clinical sample (*p* > 0.05). This indicates that adolescents in the community sample who interpreted ambiguous situations as threatening were more likely to also interpret them as hostile.

## Discussion

The current study examined whether threatening and hostile interpretations of the same ambiguous social situations were specific to social anxiety and callous-unemotional (CU) traits, respectively, and whether these relationships differed between a community and a clinical sample of adolescents. Across both samples, social anxiety was uniquely related to threatening interpretations, and CU-traits were uniquely related to hostile interpretations. Both of these relationships hold true for the community sample. However, the relationship between CU-traits and hostile interpretations was not significant for the clinical sample separately. Only adolescents with externalizing disorders scored higher on hostile interpretations. Despite these sample differences, the results overall support the content-specificity of threatening and hostile interpretation biases to social anxiety and CU-traits, respectively.

In line with our hypotheses, we found that adolescents with higher levels of social anxiety made more threatening interpretations of ambiguous situations in both the clinical and the community sample. This finding is in line with other studies that found positive associations between social anxiety and threatening interpretation biases in samples with varying levels of anxiety (e.g. [32, 33, 37, 38]). In both samples threatening interpretations increased as a function of social anxiety, and they were distinct from CU-traits. This suggests that the content-specificity hypothesis applies equally to community and clinical samples, and that threatening interpretations express themselves similarly—if not the same—in subclinical and clinical social anxiety. The only difference between the two samples was quantitative rather than qualitative. Stronger threatening interpretations in adolescents with more severe social anxiety might be explained by a stronger activation of schemas related to danger and one’s own inability to cope [3, 29, 55], as well as by more frequent negative self-statements (e.g. [3, 37]). In addition to that, the link between social anxiety and threatening interpretations is in line with many theories and recent classification systems that cluster mood-related cognitions, affect, physiology and behavior together [20, 56, 57]. Together, these results underline threatening interpretations as underlying mechanism of fear in social situations and the avoidance thereof.

Across both samples, adolescents with higher levels of CU-traits showed more hostile interpretations of ambiguous social situations. Furthermore, CU-traits were not related to threatening interpretations. This finding supports the content-specificity of hostile interpretation bias in CU-traits. However, when testing the samples separately, no significant link between CU-traits and hostile interpretations was found for the clinical sample. Only adolescents with externalizing disorders, as compared to adolescents with internalizing disorders, showed higher levels of hostile interpretation bias. This suggests that CU-traits and their cognitive correlates express themselves differently in community and clinical samples. In line with this explanation, previous research identified some variants of CU-traits which differ in terms of their comorbid problems and physiological reactivities [58, 59]. It is possible that these variants also differ in their cognitive mechanisms and interpretation biases. In line with this idea, the internal reliabilities of the self-reported Inventory of Callous-Unemotional traits (ICU) were low ranging from poor to acceptable. Thus, CU-traits were not a very reliable concept in the current study. In addition to that, the explained variance for the regression model on hostile interpretations was small. In a larger clinical sample, of which the current clinical sample was randomly extracted, we did find a significant link between CU-traits and hostile interpretation bias [47]. Together, this might suggest that the link between CU-traits and hostile interpretation bias is weak, if it exists at all, and that larger sample sizes are necessary to control for the heterogeneity of CU-traits.

Some unexpected findings need to be discussed. First, adolescents of the community sample that interpreted ambiguous social situations as more threatening also interpreted them as more hostile. More research found internalizing and externalizing cognitions to correlate with each other [46, 57, 60, 61]. In response to another person’s ambiguous behavior, it might be healthy to engage in both self-doubt (e.g. s/he doesn’t like me) and other-blame (e.g. s/he is a jerk). In the clinical sample, the different interpretation biases did not significantly relate to each other. This might indicate that with a higher degree of symptomatology, interpretation biases are specific rather than general. Another explanation for the lack of significance might be the smaller sample size of the clinical sample. However, the correlation coefficient was very small (r = 0.05). Therefore, this explanation remains speculative. Second, there was an interaction between social anxiety and sample on hostile interpretations. In the community sample, hostile interpretations appeared to increase as social anxiety increased, whereas in the clinical sample, hostile interpretations appeared to decrease as social anxiety increased. This suggests that healthy adolescents with higher levels of social anxiety sometimes react callously to handle ambiguous situations (similar to the link between anxiety and aggression; [15, 16, 62]), whereas adolescents with mental health problems and higher social anxiety react fearful. Post-hoc correlations between hostile interpretations and social anxiety did not reach significance, though, neither for the community sample, nor for the clinical sample.

Despite several strengths, the current study also has some limitations. Strengths include the comparison of a community and a clinical sample, which allowed us to examine the generalizability of interpretation biases across subclinical and clinical symptomatology. Furthermore, a simultaneous assessment of different interpretations in the same situation is crucial to disentangle (content-specific) interpretation biases. Yet, the current study is only one of a few studies that made use of such a combined approach. Alas, the data were partly collected during the COVID-19 pandemic, which considerably complicated the data collection. Since home schooling and often changing COVID measures were already intense for many teachers, parents and children, little room was left for the participation in research. This led to a smaller sample size for the community sample than anticipated. Although we had enough statistical power to answer our main research questions, the power for exploratory analyses in subsamples was restricted. Future research should examine the role of moderating variables on the link between symptoms and biases with higher sample sizes. Theory-wise it would be interesting for future research to also include measurements on (observable) behavior, such as avoidance and aggression, to investigate how different symptoms and interpretations relate to it.

## Conclusions

The current study offers further support for the content-specificity of interpretation biases in social anxiety and partly in CU-traits. By investigating whether interpretation biases are unique to specific symptoms in different samples, the underlying mechanisms of different psychopathologies can be better understood. On the long-term, this knowledge could bear crucial information for the development of tailored versus general prevention and intervention paradigms.

## Data Availability

The datasets used and analyzed during the current study, as well as part of the materials can be requested from the first author upon reasonable request.
